# Naturally Occurring Hepatitis B Virus B-Cell and T-Cell Epitope Mutants in Hepatitis B Vaccinated Children

**DOI:** 10.1155/2013/571875

**Published:** 2013-11-26

**Authors:** Yu-Min Lin, Guey-Mei Jow, Shu-Chi Mu, Bing-Fang Chen

**Affiliations:** ^1^School of Medicine, Fu-Jen Catholic University, New Taipei City 24205, Taiwan; ^2^Division of Gastroenterology, Department of Internal Medicine, Shin Kong Wu Ho-Su Memorial Hospital, Taipei 11101, Taiwan; ^3^Department of Pediatrics, Shin Kong Wu Ho-Su Memorial Hospital, Taipei 11101, Taiwan

## Abstract

To control hepatitis B virus (HBV) infection, a universal HBV vaccination program for infants was launched in Taiwan in 1984. The aim of this study was to investigate the role of B-cell and T-cell epitope variations of HBsAg and polymerase in HBV infection in vaccinated children. One hundred sixty-three sera from vaccinated children were enrolled randomly. HBV serum markers, including hepatitis B surface antigen (HBsAg) and antibodies to HBsAg (anti-HBs) and core antigen (anti-HBc), were detected by ELISA. Nucleotide sequences encoding the S and the pre-S regions of HBsAg were analyzed in all HBsAg positive sera. Five children were HBsAg positive. Sequence analysis of S, pre-S, and overlapped polymerase (P) genes showed that HBV isolates of HBsAg-positive vaccinees were variants; no G145R but G145A and other substitutions were found in the “a” determinant. Fifteen, six, and eight amino acid substitutions within B-cell and T-cell epitopes of S, pre-S, and P regions were detected, respectively. Several immune-epitope mutants, such as S45T/A, N131T, I194V, and S207N in S, were detected in all isolates. In conclusion, our results suggested that these naturally occurring immunoepitope mutants, which changed their immunogenicity leading to escape from immune response, might cause HBV infection.

## 1. Introduction

Chronic hepatitis B virus (HBV) infection is a major health problem worldwide, affecting approximately 350 million individuals. The clinical outcomes of chronic HBV infection include inactive carrier state, chronic hepatitis, cirrhosis, and hepatocellular carcinoma (HCC) [1, 2]. 

Taiwan is a hyperendemic country for HBV infection, and once as many as 15–20% of general population were chronic HBV carriers [3]. The majority of chronic HBV carriers became infected early in life while living in endemic areas, especially before the age of two [4–6]. To control HBV infection, a nationwide vaccination program was launched in 1984 [7]. This program significantly reduced the rate of persistent infection of children and the occurrences of childhood hepatocellular carcinoma and fulminant hepatitis in Taiwan [8, 9]. However, this strategy is challenged by the recent discovery where HBV mutants showed amino acid exchanges in their S region of hepatitis B surface antigen (HBsAg), which might lead to reduce or abolish the binding of vaccine-induced neutralizing antibodies [8–13]. Such vaccine escape mutants showed changes in the so-called a determinant (aa 122–148) of S region, which is assumed to be the main target for neutralizing antibodies [8]. However, the clinical significance of most of these mutants is still uncertain [14]. 

It is known that HBsAg is more variable than initially expected, especially in pre-S region. It had been demonstrated that amino acid differences occurred in the pre-S gene sequence might cause alteration of immune target sites and lead to escape from immune surveillance [15]. Besides the surface protein overlaps with the polymerase (P) protein, some pre-S and S mutations could affect the amino acid sequence of P protein. Mizukoshi et al. showed that cellular immune responses to HBV polymerase could play an important role in the clearance of viruses [16]. It is possible that the sequence variations in the pre-S and overlapped P genes would change the immune epitopes for recognition and lead to HBV infection. However, little is known so far about the nature and frequency of such mutations in HBsAg positive vaccinated children. The aim of this study was to evaluate the role of B-cell and T-cell epitope variations of S, pre-S, and polymerase in HBV infection of vaccinated children.

## 2. Patients and Methods

### 2.1. Patients

Since this is a preliminary study, in order to obtain the record of the histories of familial clustering of HBV infection and hepatitis B vaccination, samples of the children born in one hospital were obtained. One hundred and sixty-three sera of HBV vaccinated children were recruited randomly at the Shin Kong Wu Ho-Su Memorial Hospital from June 2007 to September 2009. Most of these children were outpatients and some had some medical problems such as IgE-related allergy, respiratory tract infection, or gastroenteritis. The history of familial clustering of HBV infection and hepatitis B vaccination were taken. All subjects gave consent for participating in the study. The serum samples obtained from all subjects were tested for hepatitis B surface antigen (HBsAg), hepatitis B surface antibody (anti-HBs), and hepatitis B core antibody (anti-HBc). Serum samples were divided into aliquot and kept at −80°C until testing.

### 2.2. Hepatitis Virus Markers

HBsAg, anti-HBs, and anti-HBc were tested with commercial electrochemiluminescence immunoassay kits (Elecsys HBsAg, anti-HBs, anti-HBc, and HBeAg, resp., Roche Diagnostics, GMBH, Mannheim, Germany). Anti-HBs was considered positive if the value was ≥10 IU/L and the detection limit was 2.0 IU/L.

### 2.3. Extraction of Serum HBV DNA

Serum viral DNA was extracted from 200 *μ*L serum using commercial kits (QIAamp DNA Blood Mini Kit, QIAGEN Inc., Valencia, CA, USA). The extracted DNA was used for amplification and direct sequencing of S, pre-S, and overlapped P genes as described before [17, 18].

### 2.4. Quantification and Genotyping of HBV DNA by Real-Time PCR

Quantification and genotyping of HBV DNA were performed as previously described [19]. The sensitivity of this real-time PCR method was 10^2^ copies/mL.

### 2.5. Amplification and Sequencing of the Surface, Pre-S and Overlapped P Genes

The segments of surface (549 bp, nucleotide positions 247–795, overlapping with the reverse transcriptase domain of P) and pre-S (564 bp, nucleotide positions 2828–176, overlapping with the spacer domain of P) DNA were amplified by nested PCR and sequenced as previously described [17]. The forward primers of S gene were changed into S-3, 5′-TCCTAGGACCCCTGCTCGTGTTAC-3′ for the outer set and S-5, 5′-TCTAGACTCGTGGTGGACTT-3′ for the inner set. To avoid false positivity of PCR, precautions were followed strictly.

### 2.6. Alignment and Phylogenetic Analysis

Alignment analysis and construction of phylogenetic trees were performed with the help of Biology WorkBench 3.2-CLUSTALW software program (http://workbench.sdsc.edu/).

### 2.7. Ethical Considerations

The study was performed in accordance with the principles of the Declaration of Helsinki. The study was approved by the Ethical Committee of the Shin Kong Wu Ho-Su Memorial Hospital, and the sera were sampled after obtaining informed consent from the children and their parents. Children with HBV infection and their parents were informed about these positive results.

## 3. Results

### 3.1. Characteristics of HBsAg Positive Vaccinees

Five children were detected as HBsAg positive: three are girls and two are boys. The serologic status of them was shown in Table 1; four of them were HBsAg and anti-HBc copositive, and all of them were anti-HBs negative. The history of familial clustering of HBV infection demonstrated that three (Y1, Y3, and Y5) of them (60%) were born to HBsAg positive mother. Subject Y4 was born to HBsAg negative and anti-HBs positive mother, but her father is a HBV chronic carrier. All HBsAg positive sera were subjected to viral DNA isolation and sequence analysis. Direct sequencing and phylogenetic analysis of pre-S region revealed that four were genotype Ba and one was genotype C (Table 1). The titer of HBV isolates was 5.89 × 10^5^ ~ 7 × 10^6^ copies/mL (Table 1).

### 3.2. S Mutants in Vaccinated Carriers

To elucidate whether vaccine escape mutant was the cause of HBV infection or not, the sequences of S region were analyzed. Nucleotide analysis showed that several nucleotide substitutions were detected and two (T586C and A748G) of them were found in Y1–Y4 (Table 2). Amino acid alignment showed that four children (Y1–Y4) were infected with serotype adw having a lysine (K) at aa 160 and one (Y5) was infected with serotype adr having an arginine (R) at aa 160 (Table 1). It is known that all currently available genetically engineered HBV vaccines are produced with the A2 genotype/serotype adw. In order to investigate if variation within S region affects the antigenicity of S protein, which enables the virus to escape neutralizing antibodies and infect the vaccinated children, S sequences isolated from five HBsAg positive children were compared with the reference sequence GenBank no. Z35717 (genotype A2/serotype adw). Fifteen variations within B-cell epitopes (aa 44–49, aa 122–148, and aa 160–207) and T-cell epitopes (aa 28–51 and aa 136–155) were detected among these isolates when compared with the reference sequence (Table 3). Within the “a” determinant, one (N131T) substitution was detected in all isolates and three additional variants (T126I, T143S, and G145A) were found in subject Y5 (Table 3). Four replacements (S45T/A, N131T, I194V, and S207N) within the immune epitopes were observed in all isolates. One T-cell epitope (aa 163 to 174) was kept as wild type. Nine substitutions outside the B- and T-cell epitopes were also found.

### 3.3. Immune Epitope Mapping of the Pre-S Variants

To elucidate if variation in the pre-S region caused HBV infection, the sequences of pre-S gene were analyzed. The nucleotide sequences of pre-S amplicon from five infected children were more diverse than those of S region (Table 2). Notably, amino acids within some immune epitopes (B-cell epitopes in pre-S1 region: aa 19–26, aa 32–47, aa 37–45, aa 94–105, and aa 106–117; B-cell epitope in pre-S2 region: aa 38–48; T-cell epitopes in pre-S1 region: aa 12–21, aa 21–30, aa 94–105, and aa 106–117; and T-cell epitopes in pre-S2 region: aa 21–30 and aa 29~48) were maintained as wild type among these isolates (Table 4). There were six variations within B-cell (aa 12–32, aa 27–53, and aa 72–78 in pre-S1 region and aa 1–6, aa 3–15, and aa 13-24 in pre-S2 region) and T-cell (aa 29–48 in pre-S1 region) epitopes (Table 4). Fifteen substitutions outside the B- and T-cell epitopes (Q10 K, M12T, N15S, N20S, A54E, H56N, V60A, S62A, T68I, Q82S, L84I, L85F, A90V, V172A, and L173P) were found.

### 3.4. Sequence Analysis of the P Protein

Since the HBV surface gene overlaps polymerase (P) gene, variations within the pre-S and S regions can result in amino acid changes in the spacer and reverse transcriptase (RT) domain of the polymerase antigen. The deduced amino acid sequence of the corresponding fragment of the viral polymerase was also examined (Table 5). Notably, there were many substitutions within the spacer domain of P protein. Amino acids within the RT domain of the P region were less variable. The YMDD motif, which is essential for the enzymatic activity of many RTs, was conserved. Eight substitutions within the B-cell (aa 225–250) epitope were found. T-cell epitopes (aa 412–427, aa 426–435, aa 455–463, and aa 501–516) were conserved and maintained as wild type in all isolates. Many replacements were detected in the five isolates but were not involved in either B- or T-cell epitopes.

## 4. Discussion

In this study, five of 163 HBV vaccinees have HBV infections. The possible causes of HBV transmission in vaccinees are maternal transmission, vaccine escape variants, and hypo- or nonresponse to the vaccine. Hypo- and nonresponsiveness have been shown to be associated with certain HLA types [20, 21]. Previous study showed that a DR14-DR52 association may be involved in the low immune responsiveness to hepatitis B vaccine of the Chinese population in Taiwan [20]. The haplotypes of five HBsAg positive children should be determined to provide the information if specific HLA haplotypes were involved in the HBV infection. Since the present study was cross-sectional rather than longitudinal, there were no previous data on the initial response of the children to vaccination. It is unclear whether they did not respond to vaccine or they were infected by vaccine escape mutants from the beginning of infection. In order to trace the possibility of intrafamilial transmission, phylogenetic analysis of the S gene from their parents was investigated. Two of five families had sufficient serum for isolating viral DNA. The results showed that child Y4 got infected from her father and Y5 got infected from his mother.

To investigate if HBsAg seropositivity in these pediatric subjects was caused by mutations which alter the immunoreactivity of HBsAg, we performed nested PCR and direct sequencing of S gene. We did not find the most important and best-documented G145R mutation, but we found a G145A mutation and other substitutions (T126I, N131T, and T143S) within the “a” determinant. Mutations outside the “a” determinant were frequent and tend to cluster in two regions around aa 42 to 49 and 160 to 207 in this study. The first region contains both an MHC-I-restricted T-cell epitope and a B-cell epitope, whereas the second region exhibits B-cell epitope [22–25]. Previous studies showed that changes within these regions may alter the conformation of this immunogenic determinant [26, 27]. Therefore, the occurrence of these B-cell and T-cell epitope mutants might lead them to escape the immune response resulting in HBV infection.

Less attention has been given to the presence of mutations within the pre-S in HBsAg positive vaccinees. The pre-S region has an important role in HBV replication and contains B-cell and T-cell epitopes. Previous studies showed that amino acid differences that occurred in pre-S and S genes might cause alterations of the immune target sites leading to escape from immune surveillance, or abrogate the recognition by MHC-class II-restricted T helper cells, or reduce binding affinity by MHC class I-mediated presentation of modified oligopeptides on the cell surface of hepatocytes [15, 16, 22, 28]. Grottola et al. showed that the no. of mutations of HBV DNA in the pre-S2 region before liver transplantation (LT) was significantly associated with increased risk for recurrence of HBV infection after LT despite hepatitis B immune globulin (HBIG) prophylaxis [29]. We also reported that amino acid deletions in pre-S region reduce the binding of mutant large surface proteins to anti-HBs antibodies [30]. We did not find the pre-S deletion in these five HBsAg positive children, but we found sequence variations within the pre-S region, especially in B-cell epitope of pre-S2 region. These variations might alter the entire conformation of HBV surface proteins, change their antigenicity to anti-HBs, and lead to HBV infection.

The role of HBV polymerase on HBV infection in vaccinees has not been studied ever. The observation of this study showed that substitutions within the B-cell (aa 225–250) epitope [31] were found but most of T-cell epitopes [16] within the HBV polymerase were maintained as wild type in HBsAg positive vaccinated children. Because polymerase is essential for the earliest steps in the HBV life cycle, recognition of this Ag may limit early HBV spread. Mizukoshi et al. used ten CD4^+^ T-cell epitopes within polymerase to analyze their immunological effects and suggested that HBV-specific cellular immunity against polymerase was important in viral clearance [16]. Therefore, HBV vaccine including these polymerase T-cell epitopes may be considered and designed.

The interesting finding of this study is that Y3 has no detectable anticore Ab. Previous study showed that woodchuck exposed to low woodchuck HB doses (≤10^3^ virions) may get a persistent infection without serum markers [32]. Therefore, anti-HBc negativity in HBsAg positive children may result from a low-dose infection, which is insufficient to allow maturation of protective memory.

It is known that there are ten recognized genotypes (A–J). All currently available genetically engineered HBV vaccines are produced with the A2 genotype, serotype adw. The ability of A2-derived HBV vaccines to protect against non-A2 HBV genotypes has recently been brought into question following the publication of a study of 3.7 million blood donors in the USA [33]. Stramer et al. suggested that the vaccine may be less effective for non-A2 infections [33]. In Taiwan, the distribution of HBV genotype was examined and showed that genotype B is the most predominant one (83.7%), followed by genotype C (14.8%) [34]. Recently, a Taiwanese study showed that hepatitis B immunized children born to genotype C mothers had a higher rate of breakthrough of infection than those born to genotype B mothers [35]. The authors postulated that this might be due to higher viral loads typically associated with genotype C infection in mothers. However, it cannot rule out the possibility due to the genetic difference between genotype B/serotype adw and genotype C/serotype adr. As shown in Table 3, Y5 isolate (genotype C/serotype adr) is more variable within the B-cell and T-cell epitope regions than Y1–4 isolates (genotype B/serotype adw) compared with the reference sequence of A2-derived HBV vaccines. This immune variation of genotype C might give an opportunity to escape immune attack and cause infection more easily.

## 5. Conclusions

This study demonstrated that the HBV isolates from HBsAg-positive vaccinated children had many substitutions in the nucleotide and amino acid sequences within the B-cell and T-cell epitope regions. This variability might change their humoral and cellular immunoepitopes of pre-S, S, and polymerase and lead to HBV infection. Several immune-epitope mutants, such as S45T/A, N131T, I194V, and S207N in S, were detected in all isolates and needed to explore their role in HBV infection. Furthermore, functional analysis of these B-cell and T-cell epitope mutants by ELISA and T cell proliferative response should be performed to elucidate the mechanism of HBV infection in the future.

## Figures and Tables

**Table 1:** Serologic status and serum HBV DNA in HBsAg positive vaccinated children.

Subject	Age (years)	Sex^a^	Serologic marker		Genotype/serotype^c^	Viral load^d^
			Anti-HBs^b^	Anti-HBc		
Y1	5.0	F	−	+	Ba/adw	7.0 × 10^6^
Y2	4.3	F	−	+	Ba/adw	8.5 × 10^5^
Y3	6.5	M	−	−	Ba/adw	5.89 × 10^5^
Y4	7.0	F	−	+	Ba/adw	1.49 × 10^6^
Y5	6.7	M	−	+	C/adr	3.23 × 10^6^

^a^F: female; M: male.

^b^The value below the detection limit (2.0 IU/L) was considered negative for anti-HBs.

^c^Genotype and serotype were derived from sequence alignment and phylogenetic analysis.

^d^Copies/mL.

**Table 2:** Nucleotide changes in the S and pre-S regions.

Subject	S (nt 284–774)	Pre-S (nt 2848–154)
Y1	A482C, T586C, A748G	T2910C, A2989C, T3026C, G3075A, C3100T, C3174T, G35A, T109A
Y2	T586C, A748G, T753A	T2882C, A2891G, A2906G, T2910C, C2985T, A2989C, C3050T, C3091T, A3092C, C3097A, C3174T, G35A, C46A, T147C
Y3	T586C, A748G, T753A	T2910C, C2985T, A2989C, C3050T, C3091T, A3092C, C3097A, C3174T, G35A, C46A, G97A, T147C
Y4	G316A, G372T, T586C, A748G, T753A	T2910C, C2985T, A2989C, C3050T, C3091T, A3092C, C3097A, C3174T, G35A, C46A, T147C
Y5	T312C, T367C, C400A, A783G	C2875A, G2927A, A2946G, T2958C, G3000C, C3008A, C3013A, T3031G, C3033A, T3051C, A3064G, T3066A, A3111G, C3116T, G3120A, A3186G, G6C, C7A, T31C, C52T, T109A, T150C, T154C

GenBank no. D00330 (genotype Ba/serotype adw) was used as the reference sequence for Y1–Y4. GenBank number AF223958 (genotype C/serotype adr) was used as the reference sequence for Y5.

**Table 3:** Amino acid variations in the S region compared with the reference sequence of A2-derived HBV vaccine.

Subject	B-cell epitope			T-cell epitope		Region outside the B- and T-cell epitopes
	aa 44–49	aa 122–148 “a” determinant	aa 160–207	aa 28–51	aa 136–155	
Y1	S45T	N131T	I194V, S207N	L42S, S45T	Wild type	P56Q, T57I, N59S, S64C, F85C, I110L, T114S
Y2	S45T	N131T	I194V, Y200F, S207N	S45T	Wild type	P56Q, T57I, N59S, S64C, F85C, T114S
Y3	S45T	N131T	I194V, S207N	L42A, S45T	Wild type	P56Q, T57I, N59S, S64C, F85C, T114S
Y4	S45T	N131T	I194V, S207N	L42I, S45T	Wild type	P56Q, T57I, N59S, S64C, R73L, F85C, T114S
Y5	S45A, V47T, L49P	T126I, N131T, T143S, G145A	K160R, Y161F, I194V, S207N	L42S, G43R, S45A, V47T, L49P	T143S, G145A	I110L, S113T, T114S

GenBank number Z35717 (genotype A2/serotype adw) was used as the reference sequence of A2-derived HBV vaccines.

**Table 4:** Amino acid changes in the pre-S region.

Subject	B-cell epitope				T-cell epitope			Region outside the B- and T-cell epitopes
	Pre-S1		Pre-S2		Pre-S1		Pre-S2
	aa 12–32, aa 27–35, aa 41–53, aa 72–78	aa 19–26, aa 32–47, aa 37–45, aa 94–105, aa 106–117	aa 1–6, aa 3–15, aa 13–24	aa 38–48	aa 29–48	aa 12–21, aa 21–30, aa 94–105, aa 106–117	aa 21–30, aa 29~48
Y1	N48H	Wild-type	G16R	Wild type	N48H	Wild-type	Wild-type	V60A, L85F
Y2	N48H	Wild-type	G16R	Wild type	N48H	Wild-type	Wild-type	M12T, N15S, N20S, T68I, Q82S, L84I, V172A
Y3	N48H	Wild-type	G16R	Wild type	N48H	Wild-type	Wild-type	T68I, Q82S, L84I, V172A
Y4	N48H	Wild-type	G16R	Wild type	N48H	Wild-type	Wild-type	T68I, Q82S, L84I, V172A
Y5	G27D, Q51H, S73G	Wild-type	S6T	Wild type	Wild-type	Wild-type	Wild-type	Q10K, A54E, H56N, V60A, S62A, A90V, L173P

GenBank number D00330 (genotype Ba/serotype adw) was used as the reference sequence for Y1–Y4. GenBank number AF223958 (genotype C/serotype adr) was used as the reference sequence for Y5.

**Table 5:** Amino acid changes in the P region.

Subject	Spacer domain		Reverse transcriptase domain		
	B-cell epitope aa 225–250	Region outside the B-cell epitope	T-cell epitope aa 412–427, aa 426–435, aa 455–463, aa 501–516	Region outside the T-cell epitope	YMDD
Y1	E228A	S202P, V257M, T265I, P290S, R315Q, C340S	Wild type	W499R, M553V	Wild-type
Y2	P227S, E228A	S202P, S262F, P264H, P290S, R315Q, P319T	Wild type	W499R, M553V	Wild-type
Y3	P227S, E228A	S202P, S262F, P264H, P290S, R315Q, P319T, E336K	Wild type	W499R, M553V	Wild-type
Y4	P227S, E228A	S202P, S262F, P264H, P290S, R315Q, P319T	Wild type	W499R, M553V	Wild-type
Y5	V232L, S234R, T236K, I242S, L243I, S249P	T190K, I214V, F218L, Q253R, S254T, T269A, A272T, R294G, Q305H, H306N, S314P, L321F, S340T	Wild type	L437I	Wild-type

GenBank number D00330 (genotype Ba/serotype adw) was used as the reference sequence for Y1–Y4. GenBank number AF223958 (genotype C/serotype adr) was used as the reference sequence for Y5.
